# A Comprehensive and System Review for the Pharmacological Mechanism of Action of Rhein, an Active Anthraquinone Ingredient

**DOI:** 10.3389/fphar.2016.00247

**Published:** 2016-08-17

**Authors:** Hao Sun, Guangwen Luo, Dahui Chen, Zheng Xiang

**Affiliations:** School of Pharmaceutical Sciences, Wenzhou Medical UniversityWenzhou, China

**Keywords:** rhein, pharmacological mechanism, signaling pathways, precise treatment, crosstalk network

## Abstract

Rhein is a major medicinal ingredient isolated from several traditional Chinese medicines, including *Rheum palmatum* L., *Aloe barbadensis* Miller, *Cassia angustifolia* Vahl., and *Polygonum multiflorum* Thunb. Rhein has various pharmacological activities, such as anti-inflammatory, antitumor, antioxidant, antifibrosis, hepatoprotective, and nephroprotective activities. Although more than 100 articles in PubMed are involved in the pharmacological mechanism of action of rhein, only a few focus on the relationship of crosstalk among multiple pharmacological mechanisms. The mechanism of rhein involves multiple pathways which contain close interactions. From the overall perspective, the pathways which are related to the targets of rhein, are initiated by the membrane receptor. Then, MAPK and PI3K-AKT parallel signaling pathways are activated, and several downstream pathways are affected, thereby eventually regulating cell cycle and apoptosis. The therapeutic effect of rhein, as a multitarget molecule, is the synergistic and comprehensive result of the involvement of multiple pathways rather than the blocking or activation of a single signaling pathway. We review the pharmacological mechanisms of action of rhein by consulting literature published in the last 100 years in PubMed. We then summarize these pharmacological mechanisms from a comprehensive, interactive, and crosstalk perspective. In general, the molecular mechanism of action of drug must be understood from a systematic and holistic perspective, which can provide a theoretical basis for precise treatment and rational drug use.

## Introduction

**Figure 1:**
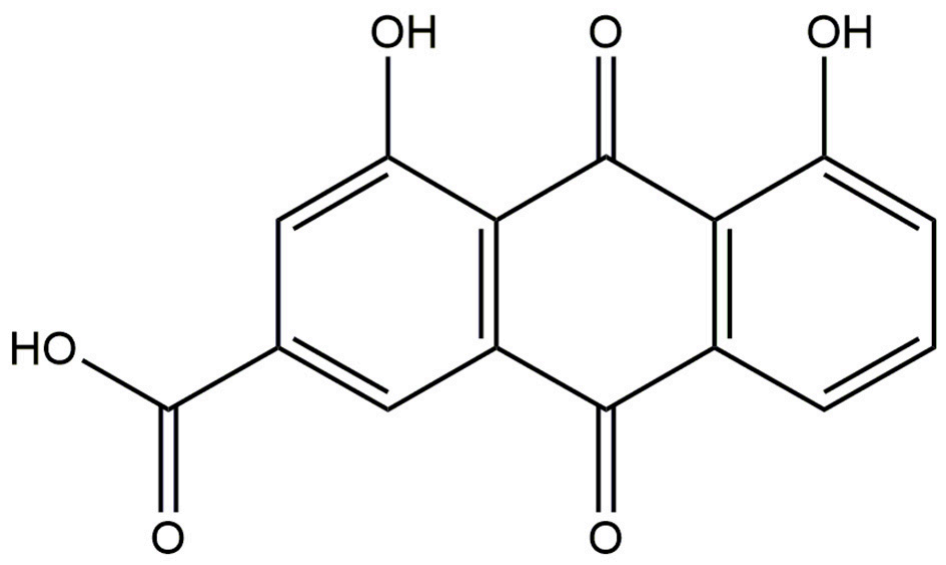
**Chemical structure of rhein**.

Rhein (Figure 1), a lipophilic anthraquinone, is an active ingredient mainly extracted and separated from several traditional rhizomes of medicinal plants, including *Rheum palmatum* L., *Aloe barbadensis* Miller, *Cassia angustifolia* Vahl., and *Polygonum multiflorum* Thunb.; rhein also has a relatively high content in *R. palmatum* L. (Nawa et al., 1961; Ge et al., 2015). *R. palmatum* L., *A. barbadensis* Miller, *C. angustifolia* Vahl., and *P. multiflorum* Thunb. have been widely used clinically for thousands of years, and they are an important part of various Chinese medicinal formulae, such as Dahuang Fuzi, Dachengqi, Heshouwu, and Yinchenhao decoction (Wang et al., 2011; Gong et al., 2012; Niu et al., 2012; Li et al., 2013). Rhein is considered the major active ingredient of these aforementioned Chinese medicinal formulae (Peng et al., 2014; Li et al., 2015). Modern pharmacological studies indicated that rhein can exert a significant therapeutic anti-inflammatory, antitumor, antioxidant, antifibrosis, hepatoprotective, and nephroprotective effects (Zhou Y. X. et al., 2015). Till date, more than 1000 articles about rhein can be found in PubMed, and more than 100 of which have paid attention on its pharmacological mechanism of action. At present, only a few articles pay attention to the relationship on the crosstalk among multiple pharmacological mechanisms. For example, rhein can significantly block ERK1/2 pathway activation (Zhu et al., 2003) and can inhibit AKT phosphorylation (Fernand et al., 2011). However, the activated AKT can inhibit Raf by the phosphorylation, thereby indirectly suppressing ERK1/2 pathway (Ersahin et al., 2015). The inhibition of AKT phosphorylation by rhein indicates that the AKT on ERK1/2 pathway inhibition is removed. Rhein regulation on the signaling pathways is the comprehensive result of the crosstalk signaling networks. Therefore, the molecular mechanism of action of drug must be understood from a systematic and holistic perspective, which can provide a theoretical basis for precision treatment and rational drug use.

In this review, we review the pharmacological mechanisms of action of rhein by consulting the literature published in the last 100 years, and these pharmacological mechanisms of action are summarized from a comprehensive, interactive, and crosstalk perspective, which can provide a valuable reference for further utilization and development of rhein. All pharmacological mechanisms of rhein are summarized in Table 1.

**Table 1:** **List of the pharmacological mechanism of rhein**.

**Pathway**	**Mechanism**	**References**
MAPK signaling pathway	Inhibiting the phosphorylation of ERK.	Martin et al., 2003, 2004; Zhu et al., 2003; Legendre et al., 2007; Lin et al., 2009; Aviello et al., 2010; Fernand et al., 2011
	Inhibiting the phosphorylation of p38 MAPK.	Lin et al., 2009; Heo et al., 2010; Hu et al., 2013
	Inhibiting the phosphorylation of JNK.	Lin et al., 2003b; Legendre et al., 2007
	Increasing the phosphorylation of ERK.	Aviello et al., 2010; Panigrahi et al., 2015
	Increasing the phosphorylation of p38 MAPK.	Lin et al., 2003a; Panigrahi et al., 2015
	Increasing the phosphorylation of JNK.	Lin et al., 2003a; Panigrahi et al., 2015
	Reducing the expression of GRB2, SOS-1 and Ras.	Lin et al., 2009
	Regulating membrane receptors and their ligands.	Kuo et al., 2004; Ip et al., 2007; Heo et al., 2010; Fernand et al., 2011; He D. Y. et al., 2011; He Z. H. et al., 2011; Su et al., 2013; Gao et al., 2014; Meng et al., 2015; Yu et al., 2015
PI3K-AKT signaling pathway	Inhibiting the phosphorylation of PI3K.	Fernand et al., 2011
	Inhibiting the phosphorylation of AKT.	Fernand et al., 2011; Cong et al., 2012a; Tsang and Bian, 2015; Wang et al., 2015
	Increasing the phosphorylation of AKT and PKC.	Panigrahi et al., 2015
	Regulating extracellular signal.	Guo et al., 2001; Gao et al., 2010; Su et al., 2013
TGF-β signaling pathway	Inhibiting the expression of TGF-β and its type I receptor.	Guo et al., 2002; Zhu et al., 2003; Zheng et al., 2008; Gao et al., 2010; He D. Y. et al., 2011; Meng et al., 2015; Tsang and Bian, 2015
	Inhibiting the effect of TGF-β on upregulating the expression of GLUT1 and stimulating the glucose uptake.	Zhang et al., 1999; Liu et al., 2001; Zheng et al., 2008
	Increasing the levels of BMP7.	Su et al., 2013
	Regulating the expression of ECM and α-SMA	Martin et al., 2003, 2004; Sanchez et al., 2003; Peng et al., 2013; Tsang et al., 2013; Tsang and Bian, 2015
Wnt signaling pathway	Increasing the expression of β-catenin, inhibiting DKK1 and DKK2.	Martel-Pelletier et al., 2011
VEGF signaling pathway	Inhibiting the expression of VEGF.	Lin et al., 2009; Fernand et al., 2011; He Z. H. et al., 2011
	Inhibiting the expression of KDR.	He Z. H. et al., 2011
	Inhibiting Hsp90α activity to induce degradation of COX-2.	Fernand et al., 2011
NF-κB signaling pathway	Inhibiting the activation or expression of NF-κB.	Lin et al., 2009; Fernand et al., 2011; Ge et al., 2011; Gao et al., 2014; Tsang and Bian, 2015; Yu et al., 2015
	Inhibiting the binding of NF-κB and AP-1.	Martin et al., 2003, 2004; Legendre et al., 2007
	Inhibiting the phosphorylation or expression of IκB.	Fernand et al., 2011; Yu et al., 2015
	Inhibiting the activation or expression of IKK.	Gao et al., 2014; Yu et al., 2015
HIF-1 signaling pathway	Inhibiting the expression of HIF-1α.	Fernand et al., 2011
AMPK signaling pathway	Increasing levels of AMPK and p-AMPK.	Panigrahi et al., 2015
	Inhibiting the expression and transcriptional activity of SREBP-1c.	Sheng et al., 2011
	Increasing the activity of CFTR.	Shi et al., 2006
FOXO signaling pathway	Increasing the activity of FOXO, upregulating the expression of Bim.	Wang et al., 2015
Cell Cycle	Regulating Cyclins.	Hsia et al., 2009; Lai et al., 2009
	Regulating CDKs.	Hsia et al., 2009
	Regulating CKIs.	Kuo et al., 2004; Ip et al., 2007; Zheng et al., 2008; Hsia et al., 2009; Lai et al., 2009; Legendre et al., 2009; Panigrahi et al., 2015
Apoptosis	Regulating TNF receptor family and its ligands.	Kuo et al., 2004; Ip et al., 2007; Deffaud et al., 2008; Heo et al., 2009, 2010; Lai et al., 2009; Gao et al., 2014; Meng et al., 2015; Yu et al., 2015
	Inducing MOMP, releasing CytC.	Bironaite and Ollinger, 1997; Lin et al., 2003a, 2007; Ip et al., 2007; Heo et al., 2009; Hsia et al., 2009; Lai et al., 2009; Li et al., 2012; Du et al., 2013; Zhao et al., 2014; Bounda et al., 2015; Panigrahi et al., 2015
	Regulating Caspase family.	Lin et al., 2003a; Ip et al., 2007; Lin et al., 2007; Shi et al., 2008; Heo et al., 2009; Lai et al., 2009; Legendre et al., 2009; Ge et al., 2011; Chang et al., 2012; Li et al., 2012; Zhong et al., 2012; Du et al., 2013; Gao et al., 2014; Zhao et al., 2014; Bounda et al., 2015; Panigrahi et al., 2015; Wang et al., 2015
	Regulating pro-apoptotic or pro-survival protein.	Lin et al., 2003a; Ip et al., 2007; Lin et al., 2007; Chipuk and Green, 2009; Gallenne et al., 2009; Heo et al., 2009; Hsia et al., 2009; Lai et al., 2009; Ge et al., 2011; Li et al., 2012; Reubold and Eschenburg, 2012; Wen et al., 2012; Zhao et al., 2014; Bounda et al., 2015; Cheng et al., 2015; Panigrahi et al., 2015
p53 signaling pathway	Increasing the expression of p53.	Kuo et al., 2004; Ip et al., 2007; Hsia et al., 2009; Zhao et al., 2014; Panigrahi et al., 2015
	Inhibiting p53.	Lai et al., 2009
Protein processing in endoplasmic reticulum	Regulating the expression of Bip.	Lin et al., 2007; Hsia et al., 2009; Lai et al., 2009; Wang et al., 2015
	Regulating the expression of CHOP.	Lin et al., 2007; Hsia et al., 2009; Lai et al., 2009; Wang et al., 2015
	Regulating ATF6, PERK, eIF2α and XBP1.	Lin et al., 2007; Cong et al., 2012a,b; Wang et al., 2015
PPAR signaling pathway	Inhibiting the expression and transcription of PPARγ.	Liu et al., 2011; Zhang et al., 2012
	Inhibiting the expression of PPARα.	Hu et al., 2011

## Rhein regulation on signal transduction

### MAPK signaling pathway

MAPK is a hub molecule of signal transduction, which is the center of various signaling pathways. MAPK can be grouped into four main families, including ERK1/2, JNK, p38 MAPK, and ERK5. ERK is mainly involved in the regulation of cell proliferation and differentiation, whereas JNK and p38 MAPK are mainly responsible for the transduction of the signal induced by stress, thereby mediating inflammation and apoptosis (Morrison, 2012). Rhein can regulate multiple sites of MAPK signaling pathways, and its targets are mainly involved in three signaling cascades, including ERK1/2, JNK, and p38 MAPK. Rhein can regulate GRB2/SOS-Ras-MAPK pathway, as shown by downregulating the expression levels of GRB2, SOS-1, and Ras, thereby resulting in the inhibition of ERK1/2 and p38 MAPK phosphorylation (Lin et al., 2009). RTKs and their ligands, as upstream regulators of GRB2/SOS-Ras-MAPK pathway, are also regulated by rhein (Fernand et al., 2011; He Z. H. et al., 2011; Su et al., 2013). Rhein can also inhibit JNK phosphorylation (Lin et al., 2003b; Legendre et al., 2007), whereas the multiple membrane receptors of JNK signaling cascade, such as Fas and TGFBR1, and the multiple signal molecules, such as Fas-L, TGF-β, TNF-α, and IL-1β, are regulated by rhein (Kuo et al., 2004; Ip et al., 2007; Heo et al., 2010; He D. Y. et al., 2011; Gao et al., 2014; Meng et al., 2015; Yu et al., 2015). Although most of the literature reported that rhein has inhibitory effects on MAPK phosphorylation, a few studies had the opposite conclusion. It is observed that a rapid phosphorylation of p38 is determined after 0.5 h when HL-60 cell was treated with 100 μM of rhein, whereas JNK phosphorylation occurs after 6–8 h (Lin et al., 2003a). Similarly, 10 μg/mL rhein significantly increases cell proliferation and ERK phosphorylation (Aviello et al., 2010).

As a matter of fact, MAPK signaling pathway is regulated by rhein in a dose-dependent manner. With small doses, rhein often plays a role in blocking the MAPK signaling pathway, while an antagonistic effect occurs at high doses. When this pathway is blocked, biological processes are usually negative at the cellular level. Thus, cells are typically characterized by reducing proliferation and secretion of inflammatory mediators. The mediation mechanism partly explains why the rhein, with small doses, shows antitumor effects and anti-inflammatory activities (Martin et al., 2003). On the contrary, high doses rhein usually shows a significant toxicity which was demonstrated to increase cell proliferation (Aviello et al., 2010).

### PI3K-AKT signaling pathway

PI3K-AKT signaling pathway widely exists in many kinds of cells, thereby regulating various cellular functions, including cell proliferation, differentiation, apoptosis, and glucose transport (Morgan et al., 2009). PI3K is an important molecule for extracellular signal transduction, and its activation is controlled by different kinds of membrane receptors and Ras protein. Various growth factors, cytokines, chemokines, extracellular matrix, or other signals activate their corresponding receptors. Then, these receptors phosphorylate themselves and activate PI3K (Hevner, 2015). PI3K activation converts PIP2 to PIP3. PIP3, a second messenger, promotes AKT protein phosphorylation through PKD1 (Morgan et al., 2009). The activated AKT regulates its downstream multiple proteins to exert various cellular functions. Rhein mainly regulates the two key proteins, namely, PI3K and AKT, to influence this signaling pathway. The majority of researchers agreed that rhein can inhibit the activation of PI3K and p-AKT, but it does not have an inhibitory effect on the total AKT (Fernand et al., 2011; Cong et al., 2012a; Tsang and Bian, 2015; Wang et al., 2015). However, rhein treatment to hepatocytes results in the increase of p-AKT1 or PKC (Panigrahi et al., 2015). In addition, several extracellular signals for this signaling pathway can also be influenced by rhein. For instance, rhein can notably increase the HGF level (Su et al., 2013) and decrease the ECM level (Guo et al., 2001; Gao et al., 2010) in the kidney tissues. The activation of PI3K-AKT signaling pathway always inhibits pro-apoptotic protein and promotes pro-survival protein (Datta et al., 1997; Cardone et al., 1998; Engelman et al., 2006). With a suitable dose, rhein always inhibits the activation of this pathway. This may be another reason for its antitumor activity, but not all of it.

### TGF-β signaling pathway

TGF-β family members, including TGF-βs, activins, and BMPs, are a multifunctional cytokine. They regulate the proliferation, differentiation, death, and migration of various cells (Moustakas et al., 2002) and influence angiogenesis, extracellular matrix regeneration, and immune suppression (Derynck and Zhang, 2003). TGF-β family member phosphorylates R-Smads through binding to the Type II receptor and recruiting Type I. Phosphorylated R-Smads combine with Smad4, and then this complex translocates into the nucleus to regulate target gene transcription (Shi and Massagué, 2003).

In many fibrosis diseases, TGF-β signaling pathway has attracted much attention. Many fibrosis-related proteins, such as ECM and α-SMA, have been expressed due to the activation of this pathway. The imbalance of ECM and overexpression of α-SMA accelerate the progression of fibrosis. Rhein can reduce FN deposition and α-SMA expression to exert its anti-fibrosis activity (Peng et al., 2013; Tsang et al., 2013; Tsang and Bian, 2015). On the contrary, some ECM components, such as COL2A1 and ACAN, are elevated by rhein (Martin et al., 2003, 2004; Sanchez et al., 2003). Moreover, rhein can inhibit the expression of TGF-β and its type I receptor (Guo et al., 2002; He D. Y. et al., 2011), antagonize the expression of GLUT1 which would be upregulated by TGF-β1, and stimulate the ability of glucose uptake in mesangial cells (Zhang et al., 1999; Liu et al., 2001; Zheng et al., 2008). In addition, rhein can also improve renal function and reduce renal fibrosis and interstitial inflammation by increasing the level of BMP7 (Su et al., 2013).

### Wnt signaling pathway

In the canonical Wnt pathway, β-catenin is a vital multifunctional protein involved in cell proliferation, differentiation, and apoptosis (Hatsell et al., 2003). The classical Wnt signal is delivered to the cytoplasm, and it eventually stabilizes the cytoplasmic β-catenin and makes β-catenin continuous accumulation. Subsequently, β-catenin transfers to the nucleus to regulate the expression of target genes (Nusse, 2005). Rhein significantly elevates the expression level of β-catenin and inhibits the Wnt antagonists DKK-1 and DKK-2 in a dose-dependent manner. This result indicates that rhein has a positive impact on osteoarthritis subchondral bone osteoblasts (Martel-Pelletier et al., 2011). It implies that the chondroprotective activity of rhein partly attributes to the regulation on this pathway.

### VEGF signaling pathway

VEGFR-2, known as KDR, is the major mediator of VEGF-driven responses in endothelial cells, and it is considered a vital signal sensor in both physiologic and pathologic angiogenesis (Cross et al., 2003). Several downstream signaling pathways are perturbed when VEGF binds to KDR. This phenomenon results in the upregulation of genes mediating the proliferation and migration of endothelial cells and promoting their survival and vascular permeability (Takahashi and Shibuya, 2005). The effect of rhein on negative regulating VEGF signaling pathway is one reason for its antitumor and anti-inflammatory activity. 20 μM rhein can downregulate the expression of VEGFA and receptor KDR to inhibit angiogenesis and cell migration completely (He Z. H. et al., 2011). Rhein can also inhibit VEGF effectively in human nasopharyngeal carcinoma cells and umbilical vein endothelial cells (Lin et al., 2009; Fernand et al., 2011). In the downstream of VEGF signaling pathway, rhein inhibits Hsp90α activity to induce the degradation of its client protein COX-2 and to promote the production of PGI2 which can inhibit the release of inflammatory mediators (Fernand et al., 2011).

### NF-κB signaling pathway

NF-κB signaling pathway involves in immunity, inflammation, apoptosis, cell survival, stress response, and other biological processes (Oeckinghaus et al., 2011). As the hub protein in this pathway, NF-κB plays a key role to regulate the gene expression induced by cytokine. Various signals initially phosphorylate IKK, and further phosphorylate IκBs. Phosphorylated IκBs are degraded by protease through ubiquitination, which activate NF-κB. The activated NF-κB subsequently enters the nucleus to combine with DNA (Perkins, 2007). IL-1β induces the degradation of IκB protein and the translocation of RELA to the nucleus; these activities are dose-dependently suppressed by rhein (Mendes et al., 2002). The binding of both NF-κB and AP-1 transcription factors can also be effectively inhibited by rhein (Martin et al., 2003, 2004; Legendre et al., 2007). As discussed under “VEGF Signaling Pathway”, rhein inhibits Hsp90α activity to induce the degradation of client protein NF-κB. IκB phosphorylation can be inhibited by rhein under normoxic or hypoxic conditions (Fernand et al., 2011). In LPS-activated macrophages, rhein inhibits NF-κB activation and sequentially suppresses the transcription of several downstream genes and inhibits NO and IL-6 levels by inhibiting IKKβ (IC50 ≈ 11.79 μM). Rhein also elevates the activity of Caspase-1 by inhibiting intracellular IKKβ, thereby increasing the release of IL-1β and HMGB1. Rhein significantly increases TNF-α secretion and the phagocytosis of macrophages with or without LPS because of IKKβ inhibition (Gao et al., 2014). In general, rhein can inhibit the activation of IκB, IKK, and NF-κB (Lin et al., 2009; Ge et al., 2011; Yu et al., 2015). Rhein exerts its antitumor and anti-inflammatory activity through the downregulation of the pathway.

### HIF-1 signaling pathway

HIF-1α, which is a key regulator of oxygen balance, plays essential roles in the angiogenesis of tumors and mammalian development (Lee et al., 2004). HIF-1α is degraded under normoxia. HIF-1α is stable only under hypoxia, and it interacts with coactivators to induce the activation of hypoxia target genes by regulating its transcriptional activity (Semenza, 2010). Rhein is a potential HIF-1α activity inhibitor in hypoxia-induced tumor angiogenesis of breast cancer cells. Rhein dose-dependently and significantly suppresses CoCl2-stabilized HIF-1α expression in MCF-7 and MDA-MB-435s cells (Fernand et al., 2011). The downregulation of the pathway is not the most important, but is essential condition for the antitumor activity of rhein.

### AMPK signaling pathway

AMPK, which is a transducer of cellular energy status, can be activated by metabolic stresses that disturb energy balance and by the upstream kinase of AMPK through phosphorylation (Hardie, 2004). The activated AMPK switches on the catabolic pathways to generate ATP, whereas it switches off the ATP-consuming processes, such as biosynthesis, cell growth, and proliferation (Towler and Hardie, 2007).

Compared with the controls, AMPK and p-AMPK in the treatment group with 50 μM rhein are increased to an extent of 2.3- and 1.6-fold in hepatocytes, respectively. AMPK can activate p53 to induce apoptosis, suggesting that rhein can lead to liver toxicity (Panigrahi et al., 2015). AMPK also can inhibit the lipogenic enzyme SREBP-1c indirectly. Gene analysis and Western blot analysis showed that rhein obviously inhibits the expression of SREBP-1c and its target genes in the liver. Luciferase reporter assay revealed that rhein suppresses the transcriptional activity of SREBP-1c through its upstream regulator LXR. These results indicate that rhein can improve nonalcoholic fatty liver disease and associated disorders through LXR-mediated negative energy balance (Sheng et al., 2011). It is interesting that, rhein regulation on the pathway shows two opposite results, liver toxicity and protection. In fact, this situation results from two parallel and independent mechanisms of AMPK downstream. However, mediation mechanisms, playing a dominant role, depends on many factors because of the complexity of biological networks. In addition, 5 μM rhein can significantly activate the downstream protein CFTR of AMPK signaling pathway, and the full activation was detected at 20 μM, thereby promoting CL^−^ secretion (Shi et al., 2006). Maybe the purgative activity of rhein is traceable to this regulatory mechanism.

### FOXO signaling pathway

FOXO regulates the expression of genes in cellular physiological events, such as cell metabolism, differentiation, cycle arrest, DNA repair, and other reactions to cellular stress (Glauser and Schlegel, 2007). FOXO activation is regulated through several posttranslational modifications, including phosphorylation, acetylation, methylation, and ubiquitylation (van der Horst and Burgering, 2007). Researchers transfected FOXO-responsive luciferase construct and Renilla luciferase reporter plasmid (pRL-TK) into MCF7 and HepG2 cells. Then, the cells were treated with or without 100 μM rhein for 24 h, and the dual luciferase activity was detected. The experiments determined that rhein can enhance the activity of FOXO in MCF7 and HepG2 cells. In addition, the expression of FOXO3-mediated Bim is induced and upregulated by rhein (Wang et al., 2015). The upregulation of the pathway is a key mechanism of the potential rhein-induced cancer cells apoptosis.

## Rhein regulation on cellular processes

### Cell cycle

In general, mitosis can be roughly divided into two stages, including interphase and mitotic stage. In cell cycle, cyclins and CDKs are the two key regulatory molecules determining a cell progress, and none of these two molecules has a catalytic activity (Nigg, 1995). These two molecules can exert kinase activity only when they form cyclin–CDK complex. In each phase, the corresponding cyclins regulate the cell cycle. For example, cyclin D1 and cyclin E regulate G1 phase to S phase; cyclin A regulate S phase to G2 phase; and cyclin B regulate G2 phase to M phase. Different cyclin–CDK combinations have different biological functions. CDK4/6 associated with cyclin D controls cell growth of G1 phase. CDK2 combined with cyclin A or cyclin E is responsible for chromosome duplication. CDK1 binds to cyclin A or cyclin B to regulate mitosis and meiosis (Lim and Kaldis, 2013). Cyclin-CDK inhibitors, such as p15Ink4b, p16Ink4a, p21Cip1, and p27Kip1, are involved in the negative regulation of CDK activity (Okamoto et al., 1995; Levkau et al., 1998; Sherr and Roberts, 1999).

Rhein induces G0/G1 arrest through the inhibition of cyclin D3, CDK4, and CDK6 and increases the levels of p21 and p53 in A-549 cells (Hsia et al., 2009). Rhein induces S-phase arrest through the inhibition of p53, cyclin A, and E in SCC-4 cells. Immunoblot analysis demonstrated that 30 μM rhein promotes the levels of p21, p27, and Chk2 but reduces the levels of cyclin B1, cyclin A, Cdc25A, and thymidylate synthase, thereby eventually leading to S phase arrest (Lai et al., 2009). Likewise, some articles also reported that rhein increases the expression levels of p21 and p27, but no effect is detected on cyclin D1 (Kuo et al., 2004; Ip et al., 2007; Legendre et al., 2009; Panigrahi et al., 2015). However, a decrease of p21 expression is found by exposing MCGT1 cells to 25 μg/mL rhein for 48 h. This effect of rhein contributes to the reversion of the diabetic phenotype of the cells (Zheng et al., 2008). As we know, p21 is a cell cycle inhibitor. The level of p21 is regulated by rhein in a positive or negative manner. On the one hand, rhein promotes the expression of p53-induced p21; on the other hand, rhein can also inhibit TGF-β-induced p21 expression. Perhaps rhein regulation on the p21 expression in the pathway is a dynamic balance between the two mediation mechanisms of p21 expression.

### Cell apoptosis

Cell apoptosis is an autonomic ordered programmed cell death to maintain homeostasis, which is controlled by serial genes. The onset of apoptosis is controlled by numerous interrelating processes, including the receptor-mediated extrinsic pathway, the mitochondrial-mediated intrinsic pathway, and the cytotoxic granule component-mediated pathway (Cullen et al., 2010; Schleich and Lavrik, 2013). The extrinsic pathway is mainly mediated by the TNF receptor family members, such as TNFR1 and Fas (Lavrik et al., 2005). The intrinsic pathway is primarily activated by nonreceptor stimuli, such as DNA damage, ER stress, metabolic stress, UV radiation, or growth factor deprivation. The main process in the intrinsic pathway is MOMP, which results in CytC release (Jeong and Seol, 2008). However, the final activation of the effector Caspases, such as Caspase-3 and Caspase-7, result in apoptosis whether in extrinsic pathway or in intrinsic pathway.

Rhein, as a potential anticancer agent, can act on multiple protein targets in apoptosis process. In extrinsic pathway, the levels of Fas and its ligand are enhanced by the induced apoptosis effect of rhein (Kuo et al., 2004; Ip et al., 2007). The specific ELISA experiment demonstrated that rhein can increase the production of sTNFR I and sTNFR II (Deffaud et al., 2008). Some studies reported that the expression of ligand TNF-α is inhibited by rhein (Heo et al., 2010; Meng et al., 2015; Yu et al., 2015). In intrinsic pathway, rhein induces the loss of mitochondrial membrane potential, CytC release from mitochondrion to cytosol, and the cleavage of Bid protein (Lin et al., 2003a). Rhein also reduces the expression of Bcl-2 and Bcl-XL and increases the expression of Bax and Bak (Heo et al., 2009). The ratio of Bax/Bcl-2 is the critical factor that determines whether apoptosis is initiated. Rhein increases the ratio of Bax/Bcl-2 by reducing the level of Bcl-2 (Lai et al., 2009; Li et al., 2012; Zhao et al., 2014). PUMA protein has a powerful proapoptotic function, which can combine all the antiapoptotic proteins, such as Bcl-2 and Bcl-XL, and can directly activate Bax (Chipuk and Green, 2009; Gallenne et al., 2009). Apaf-1 is the real core of apoptosome, which is combined with CytC, ATP/dATP, and procaspase-9 to form apoptotic activation complex, thereby activating Caspase-9 (Reubold and Eschenburg, 2012). 50 and 100 μM rhein with respective treatment to HL-7702 cells for 12 h can improve the activity of PUMA and Apaf-1 (Bounda et al., 2015). The release of AIF and ENDO-G from mitochondria to the cytoplasm leads to DNA fragmentation (Cheng et al., 2015). Rhein can induce ENDO-G and AIF release from mitochondria into the cytosol and the nucleus (Lin et al., 2007; Lai et al., 2009). Caspase family proteins play an important role in the apoptotic pathway. Caspase-8 and Caspase-10 can accept extracellular death signal and then activate their downstream Caspase-3 and Caspase-7 to induce apoptosis (Stennicke et al., 1998). They can also initiate intrinsic pathway to cleave Bid protein and then transfer and locate to mitochondrion (Kim et al., 2000). Moreover, the activated Caspase-9 and Caspase-12 can activate Caspase-3 and Caspase-7 from different pathways. Caspase-3 plays an irreplaceable role in cell apoptosis, and its main substrate is PARP, which ultimately leads to apoptosis (Wen et al., 2012). Many studies reported that rhein can activate Caspase-1, -3, -8, -9, and -12 (Lin et al., 2007; Shi et al., 2008; Chang et al., 2012; Du et al., 2013; Gao et al., 2014). However, 100 μM rhein decreases the activity of Caspase-3 and Caspase-7, with no induction for DNA fragmentation (Legendre et al., 2009). Rhein can also induce apoptosis through PARP cleavage (Lin et al., 2003a; Panigrahi et al., 2015). Obviously, the effect of rhein inducing apoptosis is a synergetic result of the extrinsic and intrinsic pathway. The two mediation mechanisms are mutually contributive, rather than mutually replaceable. Thus, rhein regulation on apoptosis is relatively stable due to the robustness and adaptability of biological networks.

### p53 signaling pathway

p53 protein can be activated by several stress signals, including DNA damage, oxidative stress, and activated oncogenes. As a transcription factor, the activated p53 initiates the cell cycle arrest, cell senescence, and cell apoptosis. The expressed protein of p53 responsive gene has an influence on many other signaling pathways in cells (Harris and Levine, 2005). Many articles reported that rhein significantly increases the expression of p53 (Hsia et al., 2009; Zhao et al., 2014; Panigrahi et al., 2015). However, it is reported that rhein induces S phase arrest by inhibiting p53 (Lai et al., 2009). This result is contrary to the present molecular mechanisms that p53 itself can induce cell cycle arrest indirectly.

## Rhein regulation on other signaling pathways

### Protein processing in endoplasmic reticulum

Endoplasmic reticulum, which is an elaborate membrane system of cells, is the main place for protein processing. Misfolded proteins that are bound to Bip are degraded through ERAD (Stolz and Wolf, 2010). The accumulation of a large number of misfolded proteins in the ER causes ER stress and activates UPR. UPR includes three parallel pathways, namely, PERK, ATF6, and IRE1 pathways. Cells do not induce apoptosis when UPR does restore the normal function of endoplasmic reticulum (Malhotra and Kaufman, 2007).

The targets of rhein are involved in the multiple sites of the three UPR pathways. The results of Western blot analysis showed that GRP 78 (also known as Bip) expression is enhanced after rhein treatment in NPC cells. The significant increase in CHOP protein levels is observed after 12 or 24 h of rhein treatment (Lin et al., 2007). The results of flow cytometric analysis also showed that rhein increases the levels of endoplasmic reticulum stress hallmarks, such as GADD153 (also known as CHOP) and GRP78 (Hsia et al., 2009). In MCF-7 and HepG2 cells, rhein-induced nonclassical UPR is further investigated. Rhein induces the expression of CHOP and the phosphorylation of eIF2α. Rhein also increases the expression of GRP78 and XBP1 splicing (Wang et al., 2015). As ER stress sensors, ATF6 and PERK are significantly induced after 6 h rhein-treatment. This result increases the susceptibility to ER stress-induced apoptosis (Lin et al., 2007). However, the effects of rhein on ATF-6 are not all positive. Rhein can also significantly inhibit ATF-6 activation to exert its anti-inflammatory activity (Cong et al., 2012a,b). In general, rhein can apparently upregulate ER stress, while the regulation on ATF-6 is controversial. Maybe the inconsistent regulation is the synthetical result of multiple upstream pathways.

### PPAR signaling pathway

PPAR is a nuclear hormone receptor, and it can be activated by fatty acids and their derivatives. PPAR has three subfamilies, which are involved in the regulation of the major metabolism, inflammation, and the important process of controlling cell fate (Feige et al., 2006). PPARα can regulate the expression of genes related to lipid metabolism in liver or skeletal muscle to clear the circulating or cellular lipids. PPARβ/δ is involved in lipid oxidation and cell proliferation. PPARγ promotes the differentiation of adipose cells to improve blood glucose absorption (Desvergne and Wahli, 1999). The effects of rhein on the differentiation of adipocytes and the lipogenesis were studied. The result showed that rhein can downregulate the expression of PPARγ and C/EBPα, which are adipogenesis-specific transcription factors, and the upstream regulator of these factors, namely, C/EBPβ, is the same. Moreover, the target genes of PPARγ involved in adipocyte differentiation are suppressed by rhein; these target genes include CD36, aP2, acyl CoA oxidase, uncoupled protein 2, acetyl-CoA carboxylase, and fatty acid synthase (Liu et al., 2011). Gene expression analysis showed that as a potential antagonist of PPARγ, rhein inhibits the transcription of PPARγ and the expression of its target genes. It indicates that rhein can blocked high-fat diet-induced obesity (Zhang et al., 2012). In addition, the derivatives of rhein can significantly alleviate diabetic nephropathy via improving the expression of PPARα in renal tissue (Hu et al., 2011). These evidence implies that rhein is an underlying candidate for preventing metabolic disorders.

## Rhein regulation on the crosstalk among pathways

**Figure 2:**
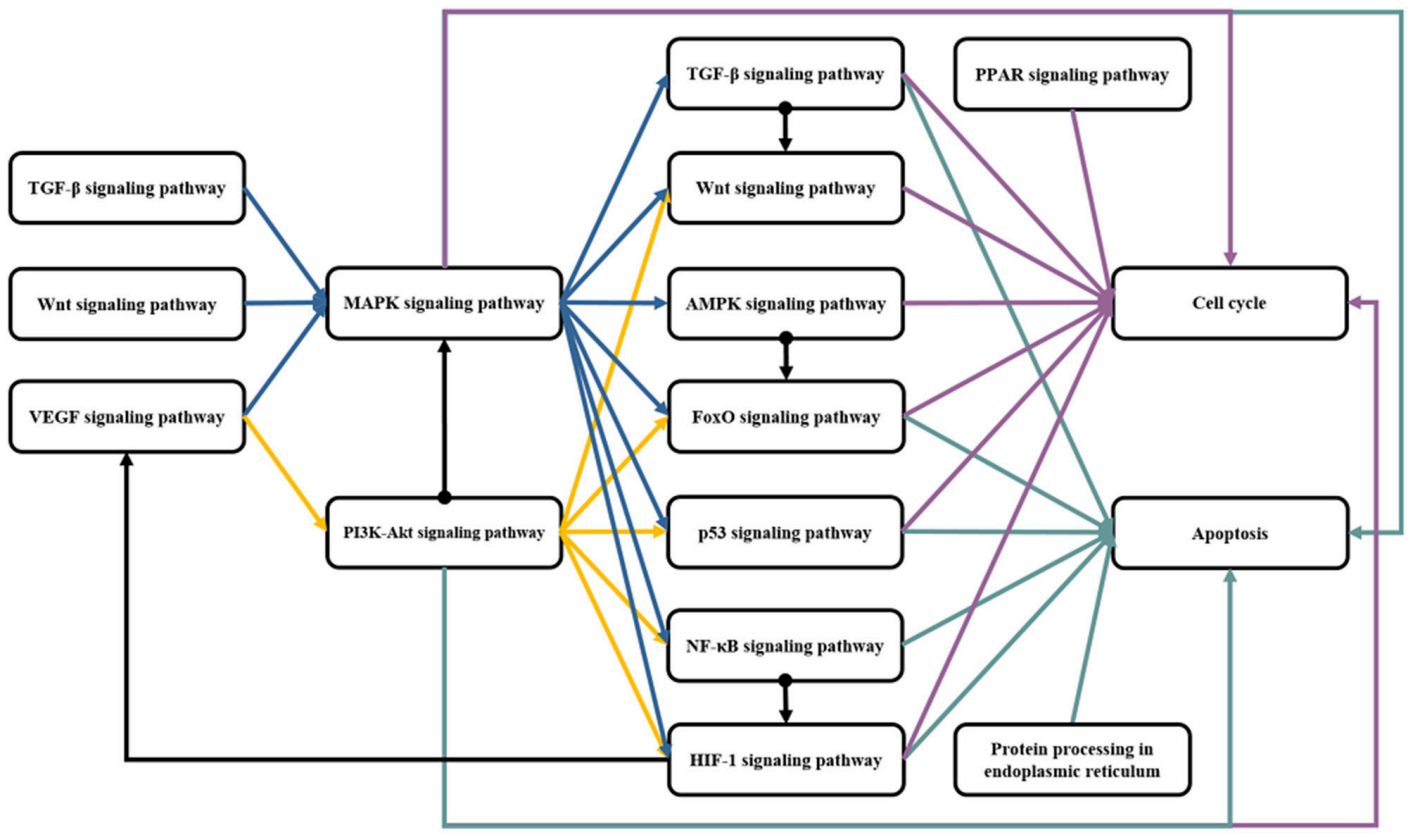
**The crosstalk among the signaling pathways related to pharmacological mechanism of rhein**. The pathways related to rhein are initiated by the membrane receptors of several pathways. Then, MAPK and PI3K-AKT parallel signaling pathways are activated, and several downstream pathways are affected, thereby eventually regulating cell cycle and apoptosis. Blue line is the crosstalk of MAPK signaling pathway; Yellow line is the crosstalk of PI3K-AKT signaling pathway; Purple line is the crosstalk of cell cycle; Green line is the crosstalk of apoptosis; Black line is the crosstalk of other pathways.

As a multitarget molecule, rhein exerts its therapeutic effect via multipathways. The pharmacological action of rhein is the comprehensive regulation result on the crosstalk among signaling pathways. As shown in Figure 2, the targets of rhein are involved in a wide range of pathways. Comprehensive crosstalk exists among these pathways.

### Crosstalk of MAPK signaling pathway

**Figure 3:**
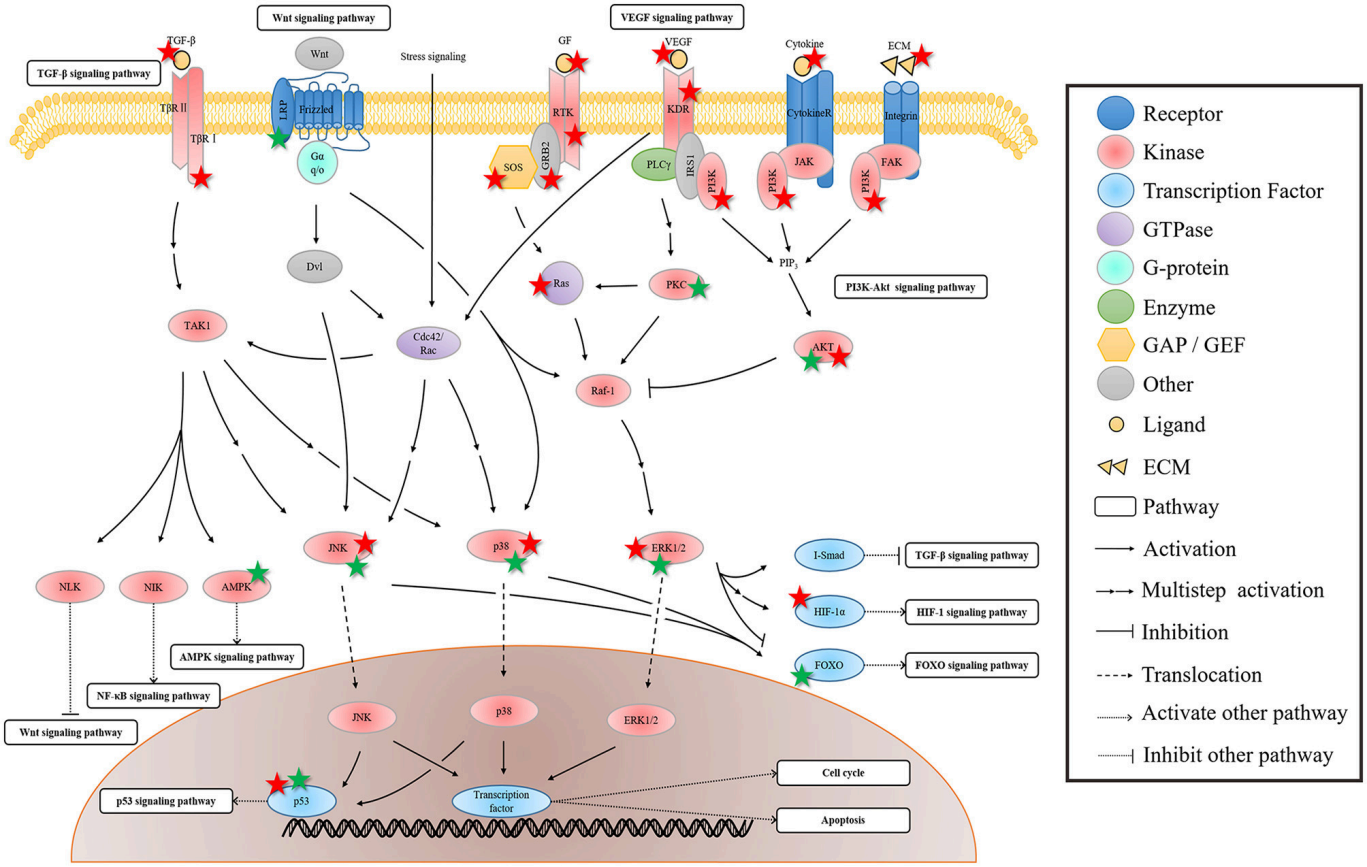
**The regulation of rhein on the crosstalk of MAPK signaling pathway**. The MAPK signaling pathway is activated by the membrane receptors of several pathways, and then its downstream pathways are regulated. Rhein acts on multiple sites of these pathways to exert the extensive and effective pharmacological activity. The protein marked with star is the target of rhein. Red and green star represent negative and positive regulation, respectively.

In the pathways involved in the pharmacological pathways of rhein, MAPK signaling pathway can be considered one of the most interactive pathways. The activation of MAPK is affected by four pathways, including VEGF, TGF-β, Wnt, and PI3K-AKT signaling pathways. Figure 3 shows that VEGF signaling pathway starts from the combination of VEGF and its receptor KDR. The activated KDR induces ERK1/2 phosphorylation by indirectly activating PLCγ-mediated PKC, rather than by depending on GRB2/SOS-Ras pathway (Shinya et al., 2015). The activated KDR can also activate Cdc42 to induce the activation of Cdc42-SAPK2/p38-MAPKAPK2/3 pathway and to promote stress fiber formation and endothelial cell migration (Lamalice et al., 2006). The TGF-β signal indirectly activates p38 MAPK and JNK via DAXX-TAK1 pathway (Vaidya and Kale, 2015). In the classical Wnt signaling pathway, Wnt3a can activate Raf-1-MEK-ERK cascade and JNK pathway (Yun et al., 2005; Bikkavilli et al., 2008). ERK, p38 MAPK, and JNK are activated by Wnt3a through G protein mediation (Zhang et al., 2014). PI3K-AKT and MAPK signaling pathways are two parallel pathways. The interaction of PI3K/AKT and Raf/MAPK/ERK1/2 pathways occurs in several different stages. This interaction can be positive or negative. The inhibitor of PI3K and AKT eliminates AKT phosphorylation and restores ERK1/2 phosphorylation. This phenomenon indicates that Raf/MAPK/ERK1/2 pathway is inhibited by AKT by directly phosphorylating Raf-1 (Zhou J. et al., 2015). In MCF-7 cells, AKT can phosphorylate Raf under high IGF-I concentration conditions. This phenomenon leads to crosstalk generation, Ras-Raf-MEK-ERK cascade blockage, and proliferation inhibition. However, the crosstalk of Akt-Raf does not exist under low IGF-I concentration (Moelling et al., 2002).

The influence of MAPK signaling pathway on other pathways is extensive and complex. Figure 3 shows that MAPK signaling pathway can affect the nine pathways related to pharmacological mechanisms of rhein. The activated TAK1 can activate NLK, whereas the classical Wnt signaling pathway is silenced through the degradation and ubiquitylation mediated by β-catenin or the phosphorylation of TCF/LEF mediated by NLK (Ke et al., 2016). The activated TAK1 can activate NIK, whereas the classical NF-κB signaling pathway is activated by NIK, NEMO, and IKKα (Neely et al., 2011). The activated TAK1 can upregulate AMPK activity and can regulate energy metabolism (Inokuchi-Shimizu et al., 2014). The activated ERK can regulate TGF-β signal by activating Smad6/7, and then R-Smad activation is inhibited (Derynck and Zhang, 2003). The activated ERK can also phosphorylate 4E-BP1, S6K, and MNK. MNK can directly phosphorylate eIF-4E (Sang et al., 2003), thereby eventually translating mRNA into HIF-1α protein. Therefore, ERK is involved in HIF-1 protein synthesis, and it regulates its transcriptional activity (Masoud and Li, 2015). FOXOs can be regulated by their upstream proteins through several posttranslational modifications, such as the phosphorylation of ERK, JNK, p38, NLK, and AMPK. Among these modifications, the effect of ERK on FOXO is inhibition (Eijkelenboom and Burgering, 2013). JNK and p38 can activate p53 by phosphorylation and then regulate p53 signaling pathway, thereby indirectly affecting cell cycle and cell apoptosis (Harper and LoGrasso, 2001). MAPK signaling pathway can directly affect cell cycle and cell apoptosis by activating multiple transcription factors, and then cell proliferation, differentiation, apoptosis, inflammation, and other cellular processes are regulated (Morrison, 2012).

The rhein action on MAPK signaling pathway was reported in many articles, but the findings were controversial. Although the majority of researchers believe that rhein inhibits the phosphorylation of ERK1/2, p38, and JNK, a few studies determined that rhein increases the activity of these proteins. Figure 3 shows that rhein is a multitarget molecule, and the result of its effect is not the change of a single pathway but a regulatory process of dynamic balance. Rhein with different concentrations also has a different effect. If the activation of the classic GRB2/SOS-Ras-ERK1/2 pathway is blocked by rhein in a certain concentration, then the other pathways may become the compensatory pathway because of the extensive interaction among pathways. Therefore, the activation of JNK and p38 induced by TGF-β, Wnt, or the other signals, is enhanced. Similarly, the uncertainty of the effect of rhein on ERK1/2 may also be the comprehensive results of rhein with a certain concentration. This observation must be further investigated through experiments. The downstream of this pathway can be regulated directly by rhein or indirectly by upstream MAPKs. Because of the uncertainty of regulation on MAPKs, rhein action on the downstream biological process is also indeterminate. Therefore, rhein is endowed with a variety of pharmacological activities due to the diversity of regulation.

### Crosstalk of PI3K-AKT signaling pathway

**Figure 4:**
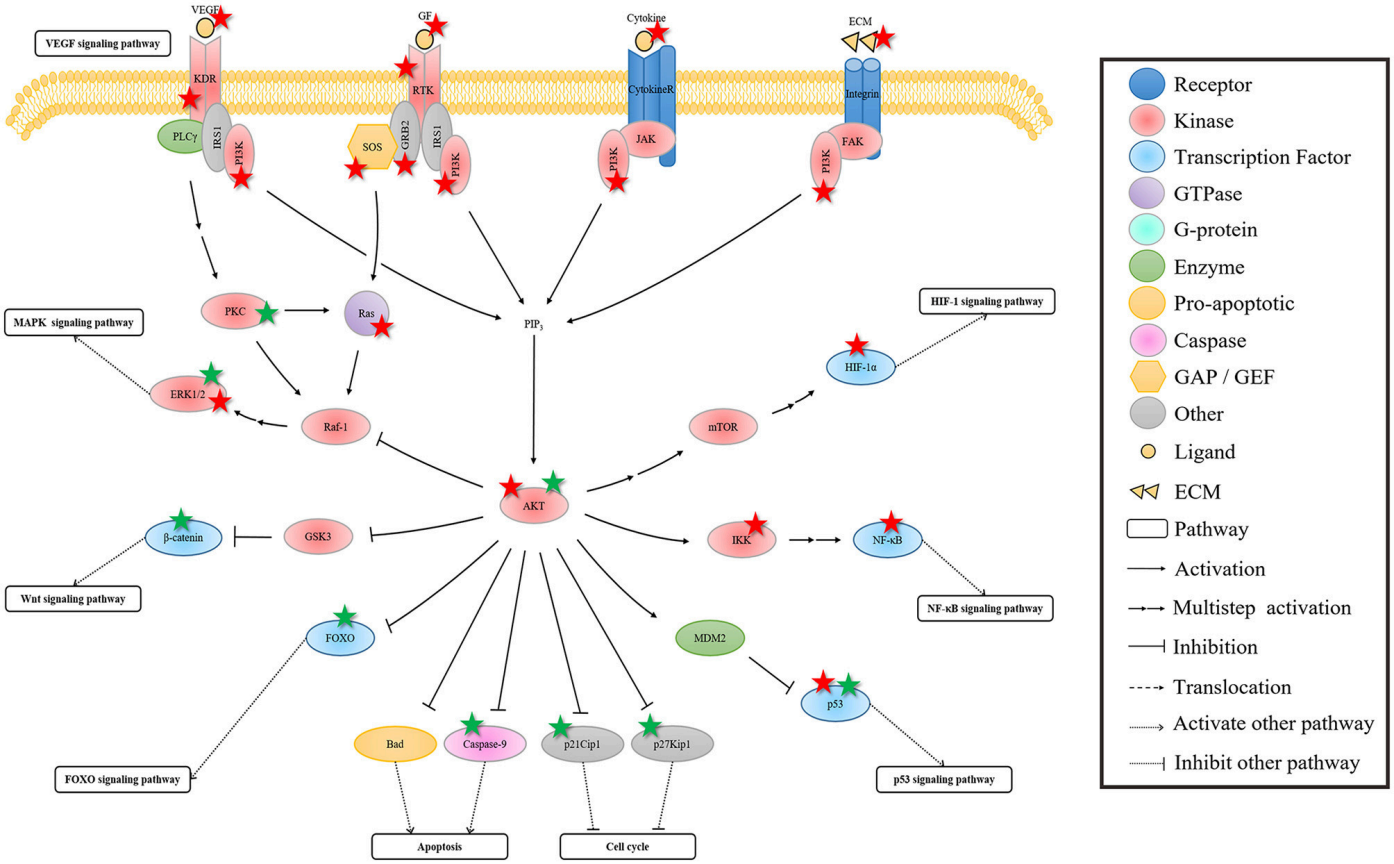
**The regulation of rhein on the crosstalk of PI3K-AKT signaling pathway**. In this pathway, AKT is the bridge of the extracellular signal and the nuclear gene expression, affecting some of its downstream pathways. The regulation of rhein on these pathways is probably because of the indirect influence of rhein through hub AKT protein mediation or the direct action to the AKT downstream protein. The protein marked with star is the target of rhein. Red and green star represent negative and positive regulation, respectively.

PI3K-AKT signaling pathway is another signal transduction pathway with a wide interaction with other signaling pathways. Figure 4 shows that this pathway can be activated by multiple membrane receptors. In addition to the MAPK signaling pathway, the activated KDR can regulate PI3K-AKT signaling pathway by activating PI3K. The activated PI3K leads to the increase of PIP3, thereby activating some important molecules, such as AKT and Rac (Cross et al., 2003).

As the hub molecule in this pathway, AKT is the bridge of the extracellular signal and the nuclear gene expression. The activation of AKT affects some of its downstream pathways. In addition to its parallel MAPK signaling pathway, Wnt, NF-κB, and FOXO signaling pathways are regulated by the activated AKT. The activated AKT can inhibit the important molecule GSK3 of Wnt signal pathway, and it then promotes Wnt signaling pathway (Cohen and Frame, 2001). GSK3 phosphorylates β-catenin when the Wnt ligand is absent, thereby resulting in β-catenin degradation, which can lead to the inhibition of Wnt signaling pathway (Jope and Johnson, 2004). The activated AKT can regulate IKK activity through direct or indirect ways (Kane et al., 1999). IKK is an upstream regulator of IκB, and it can lead to IκB degradation via phosphorylation. This series of events results in the activation of NF-κB signaling pathway. The effect of the activated AKT on HIF-1 signaling pathway is similar to the regulation of ERK on HIF-1α. AKT indirectly activates mTOR, which plays similar functions of ERK, thereby eventually activating HIF-1 signaling pathway (Richardson et al., 2004). In addition, the activated AKT can phosphorylate MDM2, thereby resulting in p53 degradation and then in p53 signaling pathway inhibition (Vivanco and Sawyers, 2002). FOXO is an important target of AKT. AKT promotes G1/S phase transition by blocking the transcription of FOXO, which mediates cell cycle inhibitor, such as p27Kip1 and RBL2 (Burgering and Medema, 2003). AKT also promotes cell survival by blocking the FOXO-mediated proapoptotic protein, such as Fas-L and Bim (Engelman et al., 2006). In fact, AKT not only indirectly affects cell cycle and apoptosis by mediating FOXO transcription factors, but also directly inhibits p27Kip1 and p21Cip1 to promote cell cycle progression in cell cycle (Engelman et al., 2006), and suppresses Bad and Caspase-9 to promote cell survival in apoptosis (Datta et al., 1997; Cardone et al., 1998).

The effect of rhein on AKT is similar to the effect on the interaction of the three MAPK cascades. The activation or inhibition of AKT may also be the result of the comprehensive effects of rhein on the PI3K-AKT signaling pathway and on its interaction pathways. In addition, rhein affects several downstream targets of AKT and some of its downstream pathways. This phenomenon occurs probably because of the indirect influence of rhein through hub AKT protein mediation or the direct action to the AKT downstream protein. Likewise, the uncertainty and variousness of rhein regulation result in the diversity of pharmacological activities.

### Crosstalk of cell cycle

As an important cellular process, cell cycle is affected by several upstream pathways. Cell cycle mainly manifests the performances for the regulation on cyclins and CDKs. By comparing the regulations of p42/p44MAPK and p38/HOGMAPK cascades on the activity and expression of cyclin D1 in CCL39 cells, researchers determined that p42/p44MAPK cascade can increase the activity and expression of cyclin D1, whereas the result of p38/HOGMAPK cascade is the opposite (Lavoie et al., 1996). The continuous activation of MAPK leads to cell proliferation when passing the restriction point of cell cycle (Fernandes et al., 1999; Hulleman and Boonstra, 2001). PI3K-AKT signaling pathway affects the transcription of cell cycle inhibitors. The activated AKT can directly or indirectly inhibit p21Cip1 and p27Kip1, thereby promoting cell cycle progression (Engelman et al., 2006). The transcription factor TCF/LEF affects cell cycle progression through the expression of cyclin D and c-myc when the classical Wnt signal is transmitted to the nucleus (Kim et al., 2013). The complexes of Smad2/3 and Smad4 in TGF-β signal pathway are transferred to the nucleus, and they increase the expression of c-myc, p15, and p21, thereby blocking cell progression (Siegel and Massagué, 2003). AMPK signaling pathway is involved in the inhibition of neural stem cell growth and cell cycle arrest by downregulating p-Rb and cyclin D (Zang et al., 2009). p53 signaling pathway can induce G1 phase arrest through p21 expression. DNA damage can activate ATM activity. Subsequently, ATM activates Chk2 via phosphorylation. Chk2 can activate the p53; it can also inactivate the Cdc25A via phosphorylation to regulate the cyclin–CDK complex (Taylor and Stark, 2001). In addition, FOXO, HIF-1, and PPAR signaling pathways can directly or indirectly interact with cell cycle to affect cell progression by regulating the expression of cyclins and their inhibitors (Harris, 2002; Fujii et al., 2004; Shao et al., 2016). Therefore, the effect of rhein on cell cycle is the comprehensive and essential regulation of multiple upstream pathways in addition to the self-regulation on cyclins, CDKs, and CKIs. In fact, rhein is always represent as the induction of cell cycle arrest while the regulation on the upstream pathways is not all consistent with it. For instance, rhein can upregulate Wnt signaling pathway, while Wnt signal can promote the expression of cyclin D and c-myc advancing cell process (Kim et al., 2013). Benefiting from the robustness of biological networks, rhein can still effectively exert its antitumor activity.

### Crosstalk of apoptosis

Apoptosis is another important cellular process. Cell cycle arrest is often accompanied by apoptosis, and apoptosis can cause cell growth arrest (Rubin et al., 1993). The level of apoptosis regulator Bcl-2 plays an important role in apoptosis. The expression and activity of Bcl-2 can be directly or indirectly affected by multiple pathways. The activation of ERK1/2 leads to the upregulation of Bcl-2 transcriptional activity. The phosphorylated Bcl-2 inhibits proapoptotic proteins and blocks the positive feedback to promote ERK1/2 activation (Liu et al., 2013). JNK and p38 are different from ERK1/2. They inhibit the expression of the prosurvival gene Bcl-2 and increase the expression of proapoptotic genes, such as p53, Fas, Fas-L, and Bim, to induce apoptosis (Chen and Chang, 2010; Hui et al., 2014; Yang and Yao, 2015). AKT can inhibit the proapoptotic gene Bad via phosphorylation to activate the prosurvival gene Bcl-2 indirectly, and phosphorylate CREB to increase the expression of Bcl-2 (Wang et al., 2016). Under hypoxia induction, HIF-1 dependent signal cascade is activated to increase the expression of Bcl-2 protein (Yang et al., 2009). Under the transcriptional control of Smad3–Smad4 signal, the upregulation of Bcl-2-interacting mediator Bim is the key of the apoptosis of Hep3B cells induced by TGF-β (Yu et al., 2008). UPR activates CHOP when the accumulation of unfolded proteins leads to endoplasmic reticulum stress, thereby inhibiting Bcl-2 (Malhi and Kaufman, 2011; Schönthal, 2012). In addition to the regulation to Bcl-2, NF-κB can express the prosurvival gene Bcl-XL to promote cell survival (Barroso-González et al., 2016). FOXO and p53 signaling pathways regulate the proapoptotic gene to promote cell apoptosis. For example, the FOXO gene can express Fas-L and Bim (Gilley et al., 2003), whereas the p53 gene can express Fas and Bax (Guo et al., 2014; Liao et al., 2016). Similar to the cell cycle, rhein directly affects multiple targets in apoptosis process and exerts the comprehensive regulation of multiple upstream pathways on the expression of apoptosis proteins. These mediation mechanisms provide a robust support for rhein to exert antitumor activity.

### Crosstalk of other pathways

In addition to the wide range of interactive pathways, such as MAPK and PI3K-AKT signaling pathways, some certain relationship exists among other signaling pathways. The Smad3/4 complex in TGF-β signaling pathway can bind to the β-catenin of Wnt signaling pathway with high affinity to block the transcriptional activity of TCF/LEF (Li et al., 2004). The crosstalk between AMPK and FOXO signaling pathways also exists. In differentiated muscle cells, AMPK can increase the content and transcription of FOXO mRNA (Nystrom and Lang, 2008). In addition, NF-κB signaling pathway promotes HIF-1 signaling pathway, which can regulate VEGF signaling pathway. In malignant lymphoma cells, the abnormal activation of HIF-1 is partly attributed to NF-κB activation (Qiao et al., 2010), whereas HIF-1 can express VEGF to promote angiogenesis in hypoxia (Chen et al., 2015). This finding further indicates that the regulation of rhein on certain pathways can affect the results of the other pathway processes.

## Rhein regulation on the whole biological network

The rhein-mediated biological network is vast and complex. As shown in Figure 2, the pathways which are related to the targets of rhein, are initiated by the membrane receptor. Then, MAPK and PI3K-AKT parallel signaling pathways are activated, and several downstream pathways are affected, thereby eventually regulating the cell cycle and apoptosis. The regulation of rhein on membrane receptors is always negative, which maybe attributes to its competitive antagonism on natural ligands. According to the extensive interaction among upstream biological networks, it is always uncertain about the regulation of rhein on hub proteins, such as MAPKs and AKT. The regulation of rhein on downstream pathways of the hub proteins is also indeterminate due to the diversity of regulation. These downstream biological processes can be regulated indirectly by the hub proteins or directly by rhein. However, the regulation of rhein is always relatively stable on the end of signal transduction, cell cycle and apoptosis because of the robustness and adaptability of biological networks.

Rhein is a multi-target molecule regulating multi-pathways at the molecular level. The therapeutic effect of rhein is the synergistic and comprehensive result of the involvement of multiple pathways. If one signal pathway is blocked in two parallel pathways, the other one may become a compensatory pathway. This phenomenon is a possible reason for the poor efficacy of single target drugs. For example, rhein can block the multiple sites of MAPK signaling pathway, thereby resulting in anti-inflammatory and antitumor effects (Martin et al., 2003). This scenario may only be a part of the explanation for the efficacy of rhein. Rhein blocks the two parallel pathways of MAPK and PI3K-AKT signaling pathways (Cong et al., 2012a), and hence it has effective anti-inflammatory and antitumor effects. In brief, the perturbation of multiple targets gives rhein a various and effective pharmacological activity, and the perturbation of a single target cannot determine the pharmacological properties of rhein.

It is notable that the impact of drug concentration on the therapeutic effect is also essential. For example, the low concentration of rhein inhibits cell proliferation by blocking MAPKs, whereas the result of rhein with high concentration is on the contrary (Lin et al., 2003a; Aviello et al., 2010). In addition, the low concentration of rhein induces the mitochondrial membrane permeability to release ROS-inducing apoptosis, whereas rhein with high concentration exerts its reducibility to inhibit ROS (Lai et al., 2009; Zhao et al., 2011).

## Conclusion

Rhein has extensive pharmacological activities, and its mechanisms are complex and interrelated. The mechanisms of rhein are involved in the multipathways which contain close interactions. The therapeutic effect of rhein is the synergistic and comprehensive result of the involvement of multiple pathways rather than the blocking or activation of a single signaling pathway. The effects of rhein with different concentrations on the pathway may vary. In this review, the pharmacological mechanisms of rhein are summarized from a systematic and holistic perspective, thereby providing an important reference for precise treatment and individualized drug administering.

## Author contributions

Conceived and designed the experiments: ZX and DC. Performed the experiments: HS, GL, and DC. Wrote the paper: HS and ZX.

### Conflict of interest statement

The authors declare that the research was conducted in the absence of any commercial or financial relationships that could be construed as a potential conflict of interest.
